# Process of inner change in advanced age: a qualitative study of older adults in their early 90 s

**DOI:** 10.1186/s12877-022-03665-5

**Published:** 2022-12-08

**Authors:** Hiroko Komatsu, Hidehito Niimura, Kaori Yagasaki

**Affiliations:** 1 Japanese Red Cross Kyushu International College of Nursing, 1-1 Asty Munakata-City, Fukuoka-Prefecture, 811-4157 Japan; 2 Faculty of Human Science, Toyo Eiwa University, 32 Miho-cho, Midori-Ku, Yokohama, 226-0015 Japan; 3 Department of Neuropsychiatry, Keio University School of Medicine, 35 Shinanomachi, Shinjuku-Ku, Tokyo, 160-8582 Japan; 4 Faculty of Nursing and Medical Care, Keio University, 35 Shinanomachi, Shinjuku-Ku, Tokyo, 160-8582 Japan

**Keywords:** Mental wellbeing, Oldest old, Longevity, Geriatric care, Qualitative research

## Abstract

**Background:**

The number of nonagenarians is growing globally. The promotion of mental wellbeing is increasingly important. The aim of this study was to explore mental wellbeing and psychological experiences of older adults in their early 90 s who were living at home.

**Methods:**

We conducted a qualitative study using semi-structured face-to-face interviews with 20 older adults in their early 90 s. A thematic analysis, according to Braun and Clarke, was used to analyze data.

**Results:**

An inner process of older adults in their early 90 s was revealed; its three themes were the “reality of aging,” “seeking emptiness of the mind,” and “still moving on.” Older adults in this study experienced functional decline, regret, and loneliness. They were tired of life and nearly gave up. Emptying their minds helped them reset their attitudes and find a way to move on. After realizing that negative thinking did not help anything, they focused on what they could do and their daily routines. Perceived social usefulness validated participants’ self-worth. However, a few were consistently active without negative perceptions of aging.

**Conclusion:**

Understanding the psychological process and mental wellbeing in later life aids in the development of practical healthcare policies to assist the growing oldest-old population in cope with age-related challenges and improve their mental wellbeing.

## Introduction

The ongoing demographic transition constitutes a challenge for healthcare providers and policymakers to help the oldest old live not only longer but also better lives by making wise use of resources [1–3]. The wellbeing of older adults is becoming increasingly important for aging individuals and society as a whole [4].

In later life, people are more likely to experience bereavement, loss of social networks, and health problems, all of which can negatively affect mental wellbeing [5, 6]. Furthermore, the potential impact of the COVID-19 pandemic on the mental health of vulnerable groups is of global concern [7, 8], particularly the oldest-old group, in which more than half of the nonagenarians reported loneliness even before the COVID-19 pandemic [9].

By contrast, a high level of mental wellbeing positively affects mortality, morbidity, and life satisfaction. Mental wellbeing has a greater impact on life satisfaction than on physical health in old age [10]. The will to live and optimism are significant predictors of survival [11, 12]. Optimism positively influences survival beyond the age of 90, regardless of rising comorbidity and independent of depression [13]. Empirical evidence suggests that mental wellbeing plays a protective role in health maintenance [3].

As the aging population is heterogeneous, the concept of segmenting the older adult group has emerged in gerontology research [14]. In Japan, where the average life expectancy (84.3 years) is the highest in the world, community-based studies of the aging population have been conducted for policy recommendations for the older population [15]. The Arakawa study is a community-based study for creating a database that would allow for detailed analysis of physical, mental, and social health [16]. Positive feelings and wellbeing were traits shared by high-functioning older adults in the Arakawa 95 + and 100 + studies. Although the need for care in the oldest old has rapidly increased, some older adults have maintained or even improved their level of care.

In our previous qualitative study, which was a part of the Arakawa study, near-centenarians, and centenarians (mean age: 98.6 years) maintained autonomy by making small decisions despite their functional decline, and found joy in the little things that made life worth living [17]. What processes do such individuals undergo to reach this perspective? This study focused on the psychological processes of older adults in their early 90 s living at home, and explored their mental wellbeing and psychological experiences.

## Methods

### Study setting and design

This study is part of the 85 + Arakawa study, a cohort study of older residents (≥ 85 years old on January 1, 2016) in Arakawa City Ward, Tokyo, Japan, with the aim of identifying contributing factors to a healthy and long life in older adults. The cohort study collected a variety of data, including physical and cognitive functions, mental health, lifestyle, and physical activities. As our previous studies [16, 17] reported positive feelings and wellbeing in older adults aged 95 years and older, this study focused on the psychological process of residents in their early 90 s. This study adopted a qualitative design and individual interviews with community-dwelling older adults were conducted. Thematic analysis was used to investigate mental wellbeing and psychological experiences of older adults in their early 90 s. This qualitative study followed the consolidated criteria for reporting qualitative research (COREQ) [18].

### Data collection

Participants were older adults who met the inclusion criteria and were purposively selected from the Arakawa cohort registry. At the three-year follow-up survey of the Arakawa 85 + study, we asked older participants aged 90–94 years to determine whether they would be interested in a qualitative in-person interview. The inclusion criteria were as follows: 1) age in their early 90 s (to differentiate from the sample in our previous study of older adults aged 95 years and older) [17], 2) those who could understand the purpose and content of the study and express their consent to participate; and 3) those who could understand the questions and responses, including those who had cognitive and/or hearing impairment. Individuals judged by a physician as inappropriate for this study, including those with significant physical deterioration, were excluded.

During the recruitment process, a research assistant called 21 candidates to explain the outline of the interview survey and asked them to participate in the study. To maintain the voluntary nature of the interviews, the 21 interviewees decided to participate in the study on their own, and visits were scheduled via telephone. One of the candidates declined from the study because of ill health a few days prior to the scheduled interview. Then, a nursing researcher (HK or KY) made home visits and explained the purpose and procedures of the study to the participants and their family members (usually a spouse or sister), both orally and in writing. Informed consent was obtained from those who agreed to participate.

An interview guide was developed for this study. We began with open-ended questions that allowed older adults to express themselves freely, including “How do you spend a typical day and what do you think about it?” “What is the same or different between now and when you were in your 80 s?” “Are you happy that you have exceeded the average life expectancy? Why?” and “What do you expect, look forward to, or worry about in your life?” Then, based on their responses, we asked exploratory questions on their mental wellbeing and psychological experiences, such as “How did you feel about it?” “Could you share your feelings and concerns?” “Does your family care about you?” and “Would you talk to me in more detail?” For relatively shorter interviews, we obtained supplementary information from the families who sat in the interview for a better understanding and deeper interpretation after the interview.

Data were collected between October 2020 and November 2021. The survey was suspended from January to August 2021 due to the COVID-19 pandemic, except in April and July. Twenty participants were interviewed at their homes. The interviews were audiotaped with permission from the participant (and family) and transcribed verbatim by a transcription service specialist. Personally identifiable information was anonymized.

### Data analysis

A thematic analysis was performed according to the following multistep process described by Braun and Clarke [19]: 1) KY independently and continuously reviewed the transcripts; 2) to generate and label initial codes, relevant data were extracted from the entire dataset; and 3) to find patterns (themes), participants’ data, and individual codes were compared and integrated. Then, candidate themes and subthemes were identified; 4) the candidate themes were reviewed to finalize them; 5) through these processes, the themes were refined, their relevance to the subthemes was confirmed, and theme meanings were clarified; and 6) quotations representing vivid examples that capture the essence of the point were selected for the results. In step 5, we ensured that the themes were not too diverse and complex, and subthemes were essentially themes-within-a-theme.

When no new analytical information emerges and the maximum amount of information on the phenomenon is provided, the data are deemed saturated [20]. Accordingly, after 20 interviews, the investigators (HK and KY) confirmed that there were no new meanings of patterns (themes) or issues of potential interest in the data, and data saturation was reached. Recruitment and data collection were completed with 20 participants.

We conducted investigator triangulation to ensure the credibility and trustworthiness of the qualitative research [21]. After completing the interim analysis of the nine interviews, the researchers (KY and HK) confirmed whether we obtained sufficient data on the participants’ mental wellbeing and psychological experiences, as well as the appropriateness of coding, analysis, and interpretation decisions. This confirmation was also made after the 18th and 20th interviews. The research team reviewed quotations, identified subthemes or emerging themes, and discussed the interpretations in a peer debriefing. KY and HK reviewed and refined all codes, subthemes, and corresponding passages for discrepancies in interpretation until a consensus was reached. To avoid placing physical and mental burdens on older adults, member checking was not performed.

## Results

Twenty older adults (11 women and 9 men) participated in this study. The mean age was 91.1 years. Table 1 summarizes their demographic characteristics. The duration of face-to-face interviews ranged from 15 to 50 min (mean: 33.1 min). Of the participants, 15 lived with family members (spouse, sister, or child), and five lived alone. Some participants used day care or visiting nursing care services.

**Table 1:** Demographic characteristics

	(n = 20)
Mean age (range)	91.1 (90–93)
Gender	
Female	11
Male	9
Living Arrangements	
Living alone	5
Living with spouse and/or others	15
Level of Dependency	
Independent	12
Support level 1^a^	4
Support level 2/ Care level 1^b^	3
Care level 2^c^	1

Level of dependency under long-term care insurance

^a^ Support level 1: almost independent

^b^ Support level 2/ Care level 1: requiring partial assistance for housework

^c^ Care level 2: requiring partial assistance for toileting

**Fig. 1:**
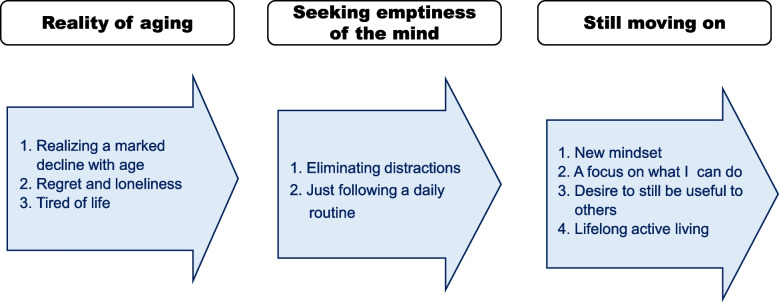
Process of inner change in advanced age

Three phases in the process of inner change among older adults in their early 90 s were identified (Fig. 1). In the first phase, participants faced the reality of irreversible aging after age 90 and felt overwhelmed by negative emotions. In the second phase, they attempted to become neutral and reset by emptying their minds. They cleared their minds of distracting thoughts and just engaged in daily routines that helped them detach from the negative perceptions of self and the future. In the third phase, they realized that life continued and they had to move on. Discovering their remaining abilities and wishes helped them to have a new mindset and take action. A few participants lived an active life with no negative perceptions about aging.

### Reality of aging

The study participants were aware of the aging process and generally experienced a decline in physical and cognitive functions, feelings of loneliness, and regrets over what they could not achieve by reviewing their lives. They were also losing the energy to find meaning in life. They were tired and seemed burdened with living.

#### Realizing a marked decline with age

Although some participants had expected to experience a decline in physical activity as they aged, the actual decline in a variety of functions made them feel that something was missing, as if they were losing who they had been:

Since my legs have stopped working, I wonder [if] this is what it is. I see. This is what happens to us as we get older. Now, I'm sad, sad. What I should say—something like, I'm stunned? (Participant M, male).

Furthermore, one of the male participants was shocked to notice a deterioration in his own cognitive and language functions, including losing track of conversations and having difficulty retrieving words:

You know, gradually, speech impediment, stammering....Even when I'm talking, I start to lose track. Sometimes I don't know what I'm talking about. That's why I was so shocked when I started to lose control of my body. I thought I was fine until then.

(Participant M, male)

Several participants realized that they had to acknowledge the reality of irreversible aging and progressive physical and functional decline when they were over 90, and they felt helpless that they had nothing to do but just grow old, as expressed below;

I've been feeling less and less strong in past years. (Participant G, male)

I'm afraid I have no choice. (Participant E, male).

#### Regret and loneliness

As the participants reached the end of their lives, many of them looked back on the past rather than to the future, expressing regret over what they had wanted to do and failed to do:

I didn't regret it until I was 80 or 90 years old. When I was younger, I didn't know anything. (Participant I, female)

Then, sad memories came to mind, and they suffered from unresolved psychological pain, as illustrated here:

Life goes by so fast. So fast. I wanted to do it right when my husband was gone. I did what I did right, but I wanted to do it better. (Participant I, female)

One of the male participants, whose house had burned three times during the war, regretted that his children had to put up with economic hardship, although he had financial responsibility for his family:

I couldn't let my child[ren] live a childlike life. I really feel sorry for them, my son and daughter. (Participant E, male)

Due to bereavement over the loss of many family members and friends, participants gradually decreased their interactions with others, experienced a greater sense of loneliness, and felt closer to their own deaths every time they lost a family member or friend, as expressed below:

I used to go out with my friends, go out drinking, but all my friends are gone. I have no friends anymore. Almost none. There are almost no people my age in the town council. They've all gone before me. (Participant E, male)

Furthermore, multiple participants felt lonely because there was no one they could talk to about their deepest feelings or who understood them. Conversations were superficial, even with their family members; the participants were often only asked about their health. They felt that they could talk to no one in depth or that no one would understand their true feelings:

Well, there are very few people I can reveal my heart [to]. (Participant I, female)

Even [when] I talk to my children…they don’t understand it. If they studied something like this [this qualitative study] or theme, it might be different. But I don’t have the chance to talk about this kind of story to my children or any other people, and they never understand me. I can only talk about specific matters, like I feel sick, I have trouble with my legs, I want you to do this….Well, no deep conversation, I think it is going to happen gradually. (Participant M, male)

Due to behavioral restrictions on COVID-19 for almost two years, one of the female participants felt regret and loneliness for not being able to say goodbye to her friends who died:

I am losing more and more friends. In these two years, three more friends passed away (…) I talked to my friends that we could have gotten together again if there were no COVID-19 pandemic (…) The impact was huge. It has been a heartless two years for older people. (Participant T, Female)

#### Tired of life

Life was long and fragile for the study participants in their 90 s. Most of them repeatedly used negative expressions such as “bother” and “sick and tired.” Their lives were narrowing in terms of independence and relationships. It was difficult to find pleasure and hope. They were negative about themselves and their future. Life seemed meaningless and wasted, as expressed below:

I can't do anything anymore. It's so boring. (Participant F, female).

What a bother! When you can't move yourself, it bothers me. (Participant D, male)

It got boring. Life is fast. It goes by before I can do anything. (Participant I, female)

I am now 93 years old, and my legs are weakening so much. I am convinced that I will not be able to walk even if I live to be 100. So, I think that it is a question of living a long life. (Participant O, female)

Some of the participants said that even daily living was a burden and that they hoped a late spouse would bring them to heaven:

I wish my late husband would come to pick me up. (Participant E, male).

It’s really annoying…I live in front of a cemetery. I visit his (my husband’s) grave and complain to him, “Why are you forgetting me (to bring to the heaven)?” (Participant C, female)

### Seeking emptiness of the mind

Many of the study participants used the terms “bother” and “sick and tired” to represent voluntarily shutting down their thoughts. In difficult situations, they tried to separate themselves from their thoughts and emotions to avoid falling into despair. They sought to empty their minds to find inner peace from nothingness. They unconsciously tried to detach themselves from the negative perceptions of self and the future by clearing their minds of distracting thoughts and concentrating on following a daily routine.

#### Eliminating distractions

Many participants did not think about their future and recognized that there was no point in doing so. Since thinking about the future could not solve their problems, they talked about the benefits of thinking about nothing and staying free of distractions:

Just so so. I feel nothing that probably good for me. (Participant A, male).

I don’t think [about the future]. Nothing starts to happen even if I think about it. (Participant J, female)

Concentrating on what they were doing also helped them dismiss their feelings of anxiety and loneliness:

When I'm gardening, I focus on that. When I have free time, I feel lonely somehow. When I'm moving, I don’t have such thoughts. (Participant L, male)

One of the participants wanted to behave like his mother because his mother had been happy until her death at the age of 103 without seriously thinking about her age and future.

No, I don’t think (about the future). In that way, my mother lived until the age of 103. So, I don’t think about age. I believe it naturally ends. I don’t think about it much. (Participant K, male)

#### Just following a daily routine

Most participants valued a routine. They spent their time in a regular routine and adopted an attitude of indifference toward other matters. This passed the time, as described below:

Regardless of being satisfied or not, what should I say? Something like passing by….Well, we are living, so we have to do so anyway. You know. (Participant E, male)

It’s annoying…everything is burdensome. I just get up in the morning and have breakfast, something like that….I have no goals. I am just living….Yes, I am just living….I just eat, drink, and sleep. That’s all. (Participant M, male)

One woman was not aware of the average life expectancy but believed that longevity was the result of a regular daily routine. Thus, she placed importance on repeating the routine, as explained here:

I have a routine that I follow almost every day, so I mostly live by that routine. I repeat it...because a year is a set number of days, right? [Life is] a series of them. It's hard to say how long we can live, and I don't have much faith in it myself. So, the age doesn’t matter. I don’t think about my age. It’s a simple routine, but I think it’s good if we repeat it. (Participant K, male)

### Still moving on

Most of the participants accepted death as a natural occurrence. They realized whether they wanted it to or not, life would go on, and thus, they had to move on. The acceptance of reality helped them take action. They were not waiting for death but striving to move forward and grow, even if only a little. They focused on what they could do, and their ultimate desire was to remain useful to others.

#### New mindset

Being older than 90 years meant that all participants were coming closer to the end of life. Some realized that death was natural and could not be resolved by resisting it. Accepting it helped ease their fear:

That's how it is with everyone. No matter how healthy you are, you'll all die in the end. (Participant T, female)

When I was young, I was scared of death and sickness. So, I thought I should try to maintain my fitness many times. Somewhere along the way, I forgot all about it. (Participant M, male)

The fear of dying is gradually disappearing. The fear of dying...I think it can’t be helped anymore. I’ve given up. It’s…something. We’re gradually giving up, aren’t we? Negative thinking helps nothing….There is no point in resisting. (Participant M, male)

Life ends someday. It is not useful to spend to maintain a negative mindset. Eventually, the participants in this study shifted to thinking positively in order to live full lives:

I have to think for myself and live by myself. Others will not help you. (Participant O, female)

I am grateful that I am healthy enough to talk like this and eat what I want and that I am not sick. (Participant P, female)

Furthermore, she continued to say the importance of the mindset:

Every day is full of life. It’s a mindset. Yes. I think it is a blessing to be able to think that way. I try to interpret things in a positive way. People who are dissatisfied are unhappy. It is better to live with satisfaction. (Participant P, female)

#### A focus on what I can do

When the participants had a new mindset, they started to actively find out what they could do. They were self-disciplined and concentrated on doing what they could, as exemplified below:

If I stay in a daze, I feel like I’m going to develop dementia, so I have to do something. I’m trying to use my head to match the numbers in Yomiuri [newspaper]. (Participant I, female).

So, to not to forget, writing and reading printed texts are something I always do. (Participant M, male)

I’m 93 years old. I guess it’s normal for my body to deteriorate like this. It's natural. On the contrary, I say, “I don’t know what I’m going to do if I don’t have strong legs.” I hold onto the refrigerator there and do 10 squats. It’s hard. I do 10 squats two times a day. (Participant O, female)

#### Desire to still be useful to others

The study participants still desired to be useful to others, even if only in a small manner. Playing their roles helped them recognize the value of their own existence. Helping others enhanced their self-esteem, motivation, and personal development, as demonstrated below:

Children and grandchildren are around. I feel they are stimulating me….When they come, (my children) bring their children….Well, it’s my role to read a picture book to them in the New Year. (Participant K, male)

First, I love flowers. People buy flowers or get small potted plants from other people and they die within a few days, but I make them last. Yes, I make them last….I make flower arrangements that are displayed somewhere special. Even if it is not particularly beautiful, when this flower blooms, I’ll cut it and give it to my friends. (Participant H, female)

One participant had been sewing since she was young. She could sense her own value because others had asked her to make masks during COVID-19, and when she did, they praised her and were pleased with her work. Such interactions with others illuminated the lives of older adults and made their lives worthwhile. She happily talked about her masks:

When I make them, everyone says, “Ms. I makes a beautiful one” and “beautiful stitch marks.” I made about 60 masks for other people. I gave them to various people. People asked me, “Did you make it?” and when I said “yes,” they said, “I want it. Please make it for me” (laugh). (Participant I, female)

#### Lifelong active living

While some participants were able to pursue active living by overcoming the negative phases, a few participants had never tired of life and were consistently active. One of them talked about her wish to travel abroad at the age of 90 after the COVID-19 pandemic.

Yes, I would like to travel abroad. It must be boring to spend time like this at home with nothing to do. (Participant H, female).

These participants not only enjoyed their hobbies but also continued to make some progress, such as learning. Thus, their lives were fulfilling, and they experienced a sense of enjoyment and hope, as expressed below:

I think that people who have hobbies can live a long life without getting bored….A man with nothing, for example, he worked for a company and retired, but working was his hobby. Such kinds of people often get sick soon after their retirement. (Participant L, male)

## Discussion

This study revealed the psychological processes of older adults in their early 90 s. Three themes emerged: “the reality of aging,” “seeking emptiness of the mind,” and “still moving on.” In their early 90 s, some older adults nearly gave up on life with negative perceptions of the self and future. Emptying their minds helped them reset their attitudes. After realizing that negative thinking did not help anything, they focused on their abilities and desires. Perceived social usefulness helped them identify their strengths and validate their self-worth.

With an increase in the oldest-old population, the focus is shifting from negative aspects, such as mortality, morbidity, and disability, to positive perspectives, such as promoting mental wellbeing [2, 6, 22]. According to the four dimensions of mental wellbeing in the oldest old defined by Lara et al. [23; functional, social, personal, and environmental], the participants of the current study had low mental wellbeing in the functional (being healthy and independent), social (social networks, interactions, engagement, and connections), and personal (personal development, being active, awareness, and personal outlook) dimensions in the first phase, the “reality of aging” of the process. Functional status still plays an important role in mental wellbeing [22]. Many participants in this study lamented their declined physical and cognitive functions, which negatively affected their mental wellbeing. Prior research reports that cognitive impairment and functional limitations are significantly associated with depressive symptoms [24].

Social engagement builds the basis of social relationships by providing a sense of belonging, social identity, and fulfillment [25]. Meaningful social roles are defined and reinforced through social activities that generate a sense of value, belonging, and attachment [26]. The participants in this study continuously experienced the loss of social networks, interactions, and connections, in addition to the loss of functionality.

Loneliness and isolation negatively affect mental wellbeing [27]. Existential loneliness emerges from physical limitations and a lack of connection with others and the world around such individuals [28]. While emotional and social loneliness stems from the absence of a significant other (close attachment) and a lack of social relationships [29], existential loneliness arises from a broader separation related to the nature of existence and a lack of meaningful existence [30]. Older adults experience existential loneliness even when they are together with other people [31] because of the absence of genuine communication [32]. The study participants who were living with family members reported that they wanted to talk about their painful experiences in the past, but could not find anyone with whom to do so. Some older adults living with their families still feel lonely because their needs are unfulfilled by their families [33]. Conversations with family members were lacking in depth, and study participants said that they were often asked only about their health conditions. Health problems may also mask other important issues in older adults [34].

A content analysis by Newall et al. [35] revealed that the most commonly reported regret among older adults was something that they had not done. Beike et al. [36] reported that people regretted lost opportunities because they could no longer change their undesired outcomes. As the number of days left for older adults is limited, their regret is likely to intensify. Sharing their regretted actions or outcomes with others may help relieve pain, although one study participant said that there were few people with whom to share their experiences and regret.

Purpose in life, which refers to having goals, a sense of direction, and meaning, is associated with improved mental and physical health outcomes [37]. Older adults are prone to think that “life is completed and no longer worth living,” [38] or that they no longer have a purpose in life after finishing their life tasks. Most participants in this study were tired of life and had experienced this phenomenon. Furthermore, some participants said that they waited for the end of their lives because they had become detached from their expectations regarding life and themselves. They want to escape from reality or even end the process [38].

In the second phase of the process, “seeking emptiness of the mind,” the participants focused on spirituality, peace of mind, and personal development in the personal dimension. When participants in this study were overwhelmed with negative thoughts and faced an identity crisis, they sought to empty their minds. Many participants tried to ignore all their distressing thoughts and shut down their thoughts because they wanted to be free from heavy psychological burdens and despair. They sought inner peace with “no distractions.” They attempted to focus on their daily routines and surrendered to the flow of life. As routines should be performed regularly, they help older adults save energy with minimal cognitive effort and perform everyday tasks, resulting in a sense of control [39, 40].

Once emptying the mind brought peace of mind, the participants realized that aging was a natural process and there was no point lamenting what was lost. They began to gain a new perspective of the world. As people age, they are likely to orient toward deeper psychological issues rather than superficial or materialistic ones [41]. Previous studies have reported a positive relationship between psychological challenges and personal growth in advanced age groups. Existential loneliness gives individuals time for self-reflection [42], and accepting age-related problems could be a sign of positive development because individuals who are aware of psychological issues are more likely to deal with them actively [34]. The participants knew that they could no longer do everything and trimmed their desires to a minimum. Living everyday is important. Regrets motivated them to seize the day and redirect their energy toward achievable goals [36]. In our previous study, the oldest old found peace and happiness after accepting their situation [17]. Thus, older adults can generally control their emotions. As perceived control implies that one can reach and control desired outcomes, it is strongly associated with a sense of wellbeing [43].

Finally, in the last phase, “still moving on,” the improvement of mental wellbeing of the oldest old was observed in all functional, social, and personal dimensions. After resetting their mindset, their personal wellbeing improved. They accepted the reality of aging, showed gratitude for life, and turned optimistic and active. They were positive and proactive in maintaining their fitness and were happy to help others. Psychological flourishing is possible with experience of purposeful life and personal growth even in very late life [44].

Social engagement is “a potential health-promoting factor” [25]. Social usefulness is an expression of a personal identity incorporated into the self that not only regulates social relationships, but also seeks opportunities for expression and verification [45]. In this study, older adults perceived rewards when they felt they were still useful to others. Self-perception was consistent with the self that other people viewed, and thus, more stable. One of the participants in this study had good sewing skills, and feedback on her handmade masks validated her self-perception. Older adults learn and grow personally through socially useful interactions, and their worth is ascribed through external validation [45]. Many older adults find value in people in their personal networks who meet their own needs for relatedness, competence, and autonomy, while also promoting others’ wellbeing [45]. High-quality networks are directly related to better mental health and possibly help compensate for declining physical health in old age [46]. As older adults often have restricted activities and short-term goals, their motivation for a socially useful identity inspires a sense of vitality and confidence [45].

The environmental dimension is described as natural surroundings and living conditions [23]. Home and neighborhood environments affect the mental wellbeing of older adults [47]. Most participants in our study lived in their own houses, and many lived with their families. Although some of the participants sometimes felt lonely because of a lack of deep communication, being with their families contributed to their mental wellbeing, which is consistent with previous studies [23, 48]. Due to COVID-19, participants’ social activities were generally restricted. They had fewer social interactions with family and friends. Only one participant, however, emphasized the negative impact of COVID-19: she was losing her friends one by one without having a chance to say goodbye. Regarding the impact of COVID-19 on mental health in older adults, a Dutch study reported increased loneliness but relatively stable mental health [7], while a French study reported that the self-rated level of mental health was low in older adults during the lockdown [49].

In this study, a few participants were consistently positive and did not experience a negative phase of the aging process. However, it remains unclear whether they underwent a different process. The differences between older adults with and without a negative phase deserve further attention in future research. As the oldest-old population is highly heterogeneous, differences across subgroups, including various levels of physical health, functional impairment, and contextual factors, should be considered [6]. Qualitative research must be embedded in a longitudinal population cohort study that includes multiple longitudinal interviews. The findings from the perspective of the oldest-old will aid in the development of practical care and support programs for this population.

This study had some limitations. First, the participants were relatively healthy older residents in a municipality in an Asian country with a long-life expectancy. Their cultural backgrounds may have affected the findings. Second, coding was conducted by one researcher (KY), not by two independent researchers. Consensus was reached by KY and HK after reviewing and refining all codes, subthemes, and corresponding passages. Third, most interviews were conducted during the COVID-19 pandemic, and lengthy interviews were not feasible. The participants might have become nervous with preventive measures such as wearing masks and maintaining social distancing. Only one participant had a 15-min interview, which was very short, and we could not obtain supplementary information from his family because he lived alone. We admit that the depth of the data is limited. In addition, comments made by family members might have influenced participants’ statements. Despite the short interview time, many participants were glad to be part of the study, talk about themselves, and help the younger generations with their experiences.

## Conclusions

The growing oldest-old population requires more resources; this is an urgent health policy agenda. Healthcare professionals and policymakers should shift away from traditional stereotype models and focus on improving wellbeing and capacities [1–3]. Physical limitations increase with age, whereas the inner self moves toward maturity, which may compensate for losses. The oldest old remain under-represented in both research and health promotion actions, especially in those that focus on mental wellbeing [2, 22]. This study provides deeper insights into the process of inner change in older adults in their early 90 s. Understanding the psychological process and mental wellbeing in later life helps develop practical healthcare policies for the growing oldest-old population to cope with age-related challenges and improve their mental wellbeing.

## Data Availability

The datasets used and/or analyzed during the current study available from the corresponding author on reasonable request. The data are not publicly available due to ethical restrictions.
